# Meta‐analysis of caries microbiome studies can improve upon disease prediction outcomes

**DOI:** 10.1111/apm.13272

**Published:** 2022-09-20

**Authors:** Mark C. Butcher, Bryn Short, Chandra Lekha Ramalingam Veena, Dave Bradshaw, Jonathan R. Pratten, William McLean, Suror Mohamad Ahmad Shaban, Gordon Ramage, Christopher Delaney

**Affiliations:** ^1^ Oral Sciences Research Group, Glasgow Dental School, School of Medicine, Dentistry and Nursing, College of Medical, Veterinary and Life Sciences University of Glasgow Glasgow UK; ^2^ R&D Innovation Haleon Weybridge UK

**Keywords:** 16S, bioinformatics, dental caries, Microbiome, sequencing, tooth decay

## Abstract

As one of the most prevalent infective diseases worldwide, it is crucial that we not only know the constituents of the oral microbiome in dental caries but also understand its functionality. Herein, we present a reproducible meta‐analysis to effectively report the key components and the associated functional signature of the oral microbiome in dental caries. Publicly available sequencing data were downloaded from online repositories and subjected to a standardized analysis pipeline before analysis. Meta‐analyses identified significant differences in alpha and beta diversities of carious microbiomes when compared to healthy ones. Additionally, machine learning and receiver operator characteristic analysis showed an ability to discriminate between healthy and disease microbiomes. We identified from importance values, as derived from random forest analyses, a group of genera, notably containing *Selenomonas*, *Aggregatibacter*, *Actinomyces* and *Treponema*, which can be predictive of dental caries. Finally, we propose the most appropriate study design for investigating the microbiome of dental caries by synthesizing the studies, which had the most accurate differentiation based on random forest modelling. In conclusion, we have developed a non‐biased, reproducible pipeline, which can be applied to microbiome meta‐analyses of multiple diseases, but importantly we have derived from our meta‐analysis a key group of organisms that can be used to identify individuals at risk of developing dental caries based on oral microbiome inhabitants.

## INTRODUCTION

Home to approximately 700 species of bacteria, the oral cavity is a complex and diverse environment and the development of oral diseases such as dental caries is closely related to the oral microbiome (1, 2, 3). Dental caries is one of the most common infectious diseases worldwide (4). The fundamental principle driving the onset and progression of dental caries stems from frequent carbohydrate intake, that causes dental plaque bacteria to produce acid, which in turn lowers the pH of the oral cavity, resulting in demineralization of the tooth surface (5). Previously thought to be primarily caused by *Streptococcus mutans*, theories about the aetiology of dental caries have developed in parallel with developments in culture‐independent techniques, and it is now considered a result of a dysbiotic oral microbiome (6, 7, 8). There remains some debate regarding the composition of the oral microbiome in caries when compared to healthy subjects, with some reporting little to no changes, whereas others state there is a shift in microbial diversity and an enrichment of pathogenic genera (9). Additionally, we recognize a fundamental gap in knowledge in relation to the interaction of more than just bacterial organisms in the oral cavity with regard to fungal species which are well documented in terms of oral dysbiosis (10, 11, 12).

The popularity of next‐generation sequencing (NGS) studies has risen hand in hand with accessibility and advances in associated technologies. This has resulted in an increase in the number of microbiome‐related studies. With this, our knowledge concerning complex areas of disease biology, such as microbiome and transcriptome, have developed rapidly in recent years. This has meant that many conditions are now hypothesized to arise because of a shift away from what is typically considered a ‘healthy’ microbiome, resulting in a more ‘dysbiotic’ community (13). However, with the ever‐increasing quantities of data that are produced from Omics studies, we are now confronted with the dilemma of how these results can be accurately and appropriately combined to enable researchers to investigate and interrogate ‘the bigger picture’ (14, 15). Indeed, the lack of standardization regarding data uploading and availability can produce difficulties in terms of methodological reproducibility and unintentional introduction of bias (16, 17). In particular, major differences in platform and sequencing region introduce high levels of variation from study to study. Despite the large number of microbiome data described within the literature, previous meta‐analysis attempts such as those carried out in the gut have found only a small subset of studies to have available or appropriate data for re‐analysis (18, 19).

By employing a meta‐analytic approach, we now have the unique opportunity to synthesize findings while addressing issues that often occur in microbiome studies such as small cohort sizes. This approach allows researchers to combine datasets and findings to identify consistencies and trends across studies, therefore helping develop our understanding of disease biology (18).

Herein, we describe a reproducible, standardized, pipeline for the downloading, processing, combination and analysis of publicly available microbiome datasets that can be applied to a myriad of health conditions. This highlights the inconsistencies in the study design of caries microbiome studies such as choice of DNA extraction kit, 16S target region and sample preparation, which can have a considerable impact on study findings.

## METHODS

### Search criteria

Studies were collected by using keyword searches on PubMed. Search results were filtered to exclude any study published before 2012 to coincide with the advancement of Illumina sequencing platforms providing more time‐effective access to microbiome analysis after this point (20). Remaining studies were exported to the reference manager EndNote (version X8). Search terms used aimed to firstly select for microbiome and NGS studies, and then, additional search terms were added using the “AND” function to further narrow search results down to those performed on the oral cavity, specifically related to dental caries.

Search results used are shown below:

((microb* OR bacteri* OR archea* OR fung* OR mycob*) AND (structure OR composition OR diversity OR community) AND (sequencing OR metabarcoding OR amplicon OR metagenom* OR 16S OR “ITS”)) AND ((“dental caries” OR “dental cavities” OR “tooth decay” OR “tooth demineralization” OR “carious dentin” OR “carious teeth” OR “caries” OR cariogenic))

### Study inclusion and exclusion criteria

All PubMed search results were exported to EndNote and upon exportation study titles and abstracts were screened for relevance and shortlisted. Studies were excluded that were not related to the oral microbiome 16 s rRNA, had no data accession number, were not mappable to individual samples, and had no additional metadata like age, sex, site and smoking status of the individuals under study. We also excluded studies with data ‘available upon reasonable request’, *in vitro* studies, transcriptomic studies and data deposited in other repositories other than the National Centre for Biotechnology Information (NCBI) or European Nucleotide Archive (ENA) database.

Shortlisted studies were interrogated in the style of a scoping review, and relevant information related to shortlisted studies was recorded by MB and BS. This relevant information included study details which may influence the microbiome or downstream data analysis, such as sampling method and site, number of cases, controls, Decayed, Missing and Filled Teeth (DMFT) index, additional cohorts, DNA extraction method, next‐generation sequencing platform, primer sequences and region of 16S rRNA. We also included the data accession number and Digital Object identifier (DOI), for further data retrieval. We then employed a two‐step approach to review shortlisted studies whereby other laboratory‐based clinicians (CLRV and SS) assessed study relevance and confirmed study detail collection. Shortlisted articles were then subjected to a scoring system where they could earn up to a maximum of 5 points. A study was awarded one point for each positive ‘yes’ to each of these questions:

1. Is the sequencing data available?
2. Is basic metadata available (*i.e*. healthy or caries sample)?
3. Can the sequencing data be mapped to the basic metadata?
4. Is additional patient metadata available?
5. Can the additional metadata be mapped to the sequencing data?

Studies were included in final analysis if they were scored as ≥3, as data access and mappability of samples to health or disease states were the minimum required criteria for further processing.

### Data retrieval and processing

Available data were downloaded from the ENA database before quality control protocols were carried out using a standardized pipeline in Qiime2 (21). Primers and barcodes were removed from reads before trimming poor‐quality reads to a minimum length of 100 bp. Paired‐end reads were merged, and then, all reads were prefiltered using sortMeRNA against the SILVA‐Bac‐16S‐id90 database (22). Filtered reads were assigned to operational taxonomic units (OTUs) by mapping to the human oral microbiome database (HOMD) and GreenGenes for downstream analysis using the Picrust databases at 97% confidence. Greengenes OTU tables were converted to a biom file before the PICRUSt normalise_by_copy_number.py and predict_metagenomes.py scripts were used to predict the functional abundancies of the microbiome for each study (23).

OTU clustering was performed closed‐reference mode using Vsearch (https://github.com/qiime2/q2‐vsearch) package within the Qiime2 (v 2019.10) analysis pipeline using the—strand both options. All representative sequences from the Qiime2 artefacts were combined before phylogenetic trees were constructed for all representative OTUs using the FastTree algorithm within Qiime2 (24).

OTU tables were exported from Qiime2 artefacts to tabular tables before both OTU tables and study metadata tables were combined and imported into R for manipulation and visualization.

### Microbiome diversity and composition analyses

We calculated sample‐based metrics upon the common Alpha diversity metrics Chao, Simpson and Shannon within a given sample (25). Alpha diversity metrics aim to apply an individual metric to each individual sample's microbiome. Chao1 gives greater weighting to low abundance calls in estimating overall species richness whereas the Simpson index is more influenced by common and dominant species. The Shannon index increases with both richness and evenness of the community. We provide 3 indexes here to avoid bias introduced by using one index over another.

Analyses were performed on all studies collectively and on a per‐study basis. The built‐in estimate_richness function in the phyloseq package (v1.30.0) was used to calculate Shannon and Simpson diversity indices and Chao1 estimates (26). Pairwise comparisons for two groups were performed by the Wilcoxon test to derive significance *p* < 0.05. For multiple groups, a Kruskal–Wallis test was performed followed by a *post hoc* Wilcoxon with a Bonferroni correction for multiple testing. All samples within the case–control studies were normalized to the geometric mean of the caries health group, and significance was determined using a Welch's t‐test. Additionally, the mean Log fold change was also calculated for each of the disease compared to health for visualization. Next, Beta‐diversity indices were employed to infer dissimilarity between samples in terms of direct distance (Euclidean), presence, absence and abundance (Bray) and phylogenetic distance (Unifrac). Matrixes were produced to estimate the diversity between each individual sample before being ordinated into the two dimensions which provided the greatest level of variation. Beta‐diversity distances were also calculated in phyloseq using the distance functions. The Centred Log Ratio (CLR) Euclidean distance matrix was calculated using the make.CLR function within MicrobeR with replacement of 0 counts using the zCompositions function and then calculating the distance matrix in base R. The phylogenetic isometric log ratio transformation (PhiLR) from the phylogenetic trees created as described above, using the R package philr (27). Distances are then calculated in base R using the dist function as described.

The function ape::PCoA was then used to ordinate the distance matrixes into a 2D plot (28). Ggplot2 was used for visualization and combining of plots within R. Permutational multivariate analysis of variance was performed on the beta diversity distance matrix which was assessed for statistical significance using the ADONIS function within the vegan package (https://github.com/vegandevs/vegan); this was performed individually on all distance matrix with 999 replications.

### Random forest classifiers

Adapting methods developed by Chen *et al* in 2021., 4 high‐impact case‐controlled studies were selected as training datasets for random forest classifiers (15). This was determined based on highest sample cohort, highest number of citations and most clearly defined sample data. The test set was comprised of the remaining studies. An additional training set was produced by randomly splitting the data in 80:20 ratio to create a training and validation data set. Centre log ratio (CLR) normalized OTUs were used as predictor variables for healthy/disease cases. OTU's table were also summarized to Genus and Species levels using the taxonomy. CLR normalized features from the PICRUSt derived KEGG orthology (KO) feature abundances and PhiLR abundances were additionally used within the random forest classifier.

Random forest models were built using the randomforest package, and receiver operator characteristic (ROC) curves were built based on random forest models using the pROC and ggROC packages (29, 30). Prediction and performance metrics were extracted using the predict and performance functions from ROCR (31). The training set was derived from subsets of the case‐controlled ‘caries vs health’ studies. Performance metrics were derived from each of the subsets of data in the remaining case‐controlled studies or the caries only studies. The most important features were extracted from random forest models by ranking MeanDecreaseGini scores and plotted using ggplot2. 10‐fold cross‐validation was used to determine the most optimal number of features from random forests built on CLR. Normalized OTUs from the PICRUSt derived database were then used to inform on the functionality of microbiome datasets and the most important features of this model were used to identify enriched metabolic pathways by matching to the KEGG database using the clusterProfiler package in R (32). Random forest models based upon the random assignment 80:20 ratio for 80% training and 20% validation were built iteratively for each individual case‐controlled study. The area under the curve was produced using the predict and performance functions for assessment of each study.

### Statistical analysis

All statistical analyses and graph production were performed using the appropriate functions in R (4.1.2) unless stated otherwise. Statistical significance was determined when *p* < 0.05.

## RESULTS

### Review of eligible studies

**FIGURE 1:**
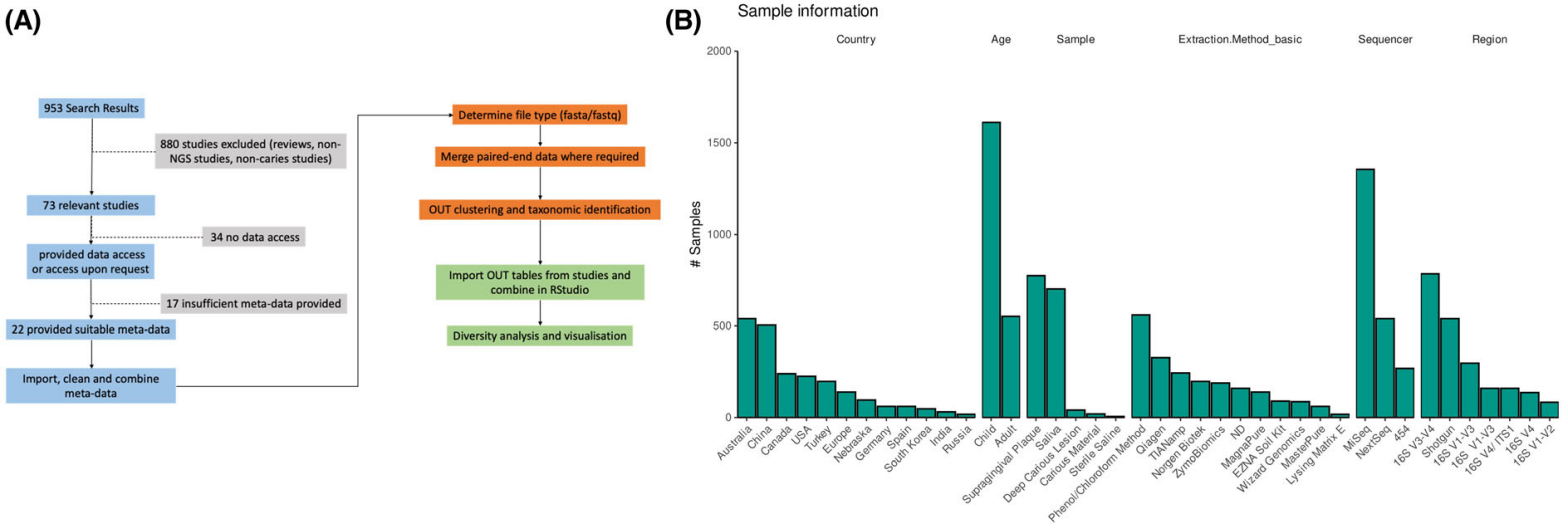
Study curation, metadata processing and study characteristics. A) A total of 623 studies were assessed for inclusion, of which 22 suitable studies were selected for further analyses. B) Breakdown of samples represented as a histogram of total samples for country, age, sample source, extraction method, platform and region sequenced. Sample data provide an indication of overlaps in interest across studies. Childhood caries was more frequently sampled than adult. Supragingival plaque and saliva are the most common sample types. Chloroform based extraction methodologies represent the largest proportion of extracted DNA. Illumina Miseq was the most popular platform by sample, and the V3‐V4 site of 16SrRNA was the most prevalently sequenced region.

First, our initial search on PubMed® produced 953 results, of which 880 were screened out based on the following exclusion criteria: review studies with no accessible sequencing data, studies not in English or not readily translatable, studies that did not specifically focus on 16S microbiome sequencing, and non‐caries‐based research. These were then assessed and scored based on data accessibility using the criteria questions outlined in our methodology and Figure 1.

In brief, it was found that 37 studies had provided no accessible sequencing data, and as such were given a 0 grading for data access. Thirty‐two studies provided access to data through publicly accessible means but had provided no means to map the samples and disease or health conditions together and were scored as 1. Eight studies provided data access and identified cohort data in the published text, usually in the form of percentage of samples associated with health or disease, but again had no means to map samples to data and received a score of 2.

Six (6) studies provided access to sample data that was clearly associated *via* sample identifiers to patients with either caries or health, and were classified as data score 3, that is the minimum criterion from the meta‐analysis. A further 7 studies provided mappable data with additional cohort data, such as age, sex and sample site, but could not match additional cohort metadata to samples and were classed as data score 4. Finally, 17 publications provided all previous criteria mentioned, with the added capacity to match additional cohort metadata to samples, receiving the highest data score of 5. This resulted in 30 publications with data access above the minimum criteria.

Further scrutiny of these 30 publications was performed, where 11 of these were then excluded based on data quality (improperly uploaded or corrupted data files), low sample number (<5 samples sequenced), sequenced region (Not individual 16S rRNA regions) and *in vitro* as opposed to *in vivo* samples. For example, 5 were not case controlled and used a ‘disease only’ cohort. This resulted in 2016 total processable samples from 19 studies for final analyses, the details of which are outlined in Table 1. These studies were initially analysed with respect to basic factors associated with study design and sample processing. Figure 1 illustrates some variability in study characteristics, location and methodologies across this cohort of 19 combined studies. When sorted by sample number, Australia was the most prominent location for caries led 16S microbiome analysis. Additionally, more than two thirds of the overall sample population were taken from children. Supragingival plaque and saliva were the two most favoured sampling sites. Illumina was the preferred sequencing platform with majority of samples being processed *via* Illumina MiSeq. The most prominent variability was observed in the sequenced region of the 16S gene, though the V1‐V3 and V3‐V4 regions were favoured. Moreover, the DNA extraction methods varied significantly from study to study, with chloroform extraction being the most favoured methodology.

**TABLE 1:** Study design and characteristics of 19 publications analysed

Publication	Location	Study design	Controls	N_Controls	Cases	N_Cases	Extraction method	Forward primer	Reverse primer	Sequencer	Region	Data accession
Jiang, 2019	Chongqing, China	Cross‐Sectional	Caries Free	22	Active Caries	24	EZNA Soil Kit	338F (5’‐ACTCCTACGGGAGGCAGCAG‐3′)	806R (5’‐GGACTACHVGGGTWTCTAAT‐3′)	MiSeq	16S V3‐V4	PRJNA495719
Zhu, 2018	Beijing, China	Cross‐Sectional	Early Childhood Caries Free	15	Early Childhood Caries Recurrence	13	QIAamp DNA Mini Kit	336F (5’‐GTACTCCTACGGGAGGCAGCA‐3′)	806R (5’‐GTGGACTACHVGGGTWTCTAAT‐3′)	MiSeq	16S V3‐V4	PRJNA493618
Kashimoglu, 2020	Istanbul, Turkey	Cross‐Sectional	Monozygotic twins	49	Dizygotic twins	50	Saliva DNA Isolation Kit	Bakt_341F (5’‐CCTACGGGNGGCWGCAG‐3′)	Bakt_805R (5’‐GACTACHVGGGTATCTAATCC‐3′)	MiSeq	16S V3‐V4	PRJNA613586
Xiao, 2016	Shanghai, China	Cross‐Sectional	Caries Free	29	Low‐High Caries	131	TIANamp Bacteria DNA Kit	8F (5’‐AGAGTTTGATCCTGGCTCAG‐3′)	533R (5’‐TTACCGCGGCTGCTGGCAC‐3′)	454	16S V1‐V3	PRJNA325084
Havsed, 2021	Jönköping, Sweden	Cross‐Sectional	Caries Free	20	Caries	20	MagNa Pure LC DNA Isolation kit II	341F (5’‐CCTACGGGNGGCWGCAG‐3′)	805R (5’‐GACTACHVGGGTATCTAATCC‐3′)	MiSeq	16S V3‐V4	PRJNA681486
Cherkasov, 2018	Orenburg, Russia	Cross‐Sectional	Caries Free (Asthma)	8	Asthma with Caries	10	Tissue Lyser LT	S‐D‐Bact‐0341‐b‐S‐17	S‐D‐Bact‐0785‐a‐A‐2	MiSeq	16S V3‐V4	PRJNA495738
Bong‐Soo, 2017	Chuncheon, South Korea	Longitudinal	Caries Free	27	Dental Caries (4 years)	12	Fast DNA SPIN extraction kit	ND	ND	454	16S V1‐V3	PRJEB19674
Gomez, 2017	California, USA	Cross‐Sectional	Caries Free	382	Dental Caries	247	Qiagen PCR purification kit	515F (5’‐GTGCCAGCMGCCGCGGTAA‐3′)	806R (5’‐GGACTACHVGGGTWTCTAAT‐3′)	MiSeq	16S V4	PRJNA383868
De Jesus, 2020	Manitoba, Canada	Cross‐Sectional	Caries Free	40	Severe early chilhood caries (SECC)	50	ND	ND	ND	MiSeq	16S V4/ ITS1	PRJNA555320
Kalpana, 2020	Tamil Nadu, India	Cross‐Sectional	Caries Free	15	Severe/Early childhood caries	40	QIAamp DNA microbiome Kit	341F (5’‐CCTACGGGNBGCASCAG‐3′)	805R (5’‐GACTACNVGGGTATCTAATCC‐3′)	MiSeq	16S V3‐V4	PRJNA454811
deJesus, 2021	Manitoba, Canada	Cross‐Sectional	Caries Free	40	S‐ECC	40	QIAamp DNA mini kit	515F (5’‐GTGCCAGCMGCCGCGGTAA‐3′)	806R (5’‐GGACTACHVGGGTWTCTAAT‐3′)	MiSeq	16S V4	PRJNA555320, PRJNA714139
Simon‐Soro, 2018	Glasgow, Scotland	Longitudinal	Caries Free (Baseline‐Follow‐Up)	14	Caries/Developed Caries (Baseline‐Follow‐Up)	19	MasterPure DNA isolation kit	8 F (5’‐AGAGTTTGATCMTGGCTCAG‐3′)	533 R (5’‐GCCTTGCCAGCCCGCTCAGGC‐3′)	454	16S V1‐V3	PRJNA421952
Xu, 2018	Beijing, China	Longitudinal	Caries Free	19	Active Caries	10	Wizard Genomic DNA Purification Kit	ND	ND	MiSeq	16S V3‐V4	PRJNA480252
Zheng, 2017	Chengdu, China	Longitudinal	Caries Free pre/post arginine	42	Caries pre/post arginine	42	TIANamp Bacteria DNA Kit	27F (5’‐AGAGTTTGATCCTGGCTCAG‐3′)	338R (5’‐TGCTGCCTCCCGTAGGAGT‐3′)	MiSeq	16S V1‐V2	PRJNA339212
He, 2017	Sichuan, China	Cross‐Sectional	Caries Free	12	Caries	25	QIAamp DNA Mini Kit	F515 (5’‐GTGCCAGCMGCCGCGG‐3′)	806R (5’‐GGACTACHVGGGTWTCTAAT‐3′)	MiSeq	16S V4	PRJNA330533
Onyango, 2020	Ghent, Belgium	Interventional	Healthy	11	Post‐treatment	11	ND	341F (5’‐CCTACGGGNBGCASCAG‐3′)	785R (5’‐GACTACHVGGGTATCTAATCC‐3′)	MiSeq	16S V3‐V4	PRJNA601417
Premaraj, 2020	Nebraska, USA	Cross‐Sectional	NA	0	Caries (between Ethnic groups)	96	QIAamp DNA mini kit	341F (5’‐CCTACGGGNBGCASCAG‐3′)	785R (5’‐GACTACHVGGGTATCTAATCC‐3′)	MiSeq	16S V3‐V4	PRJNA555622
Ferrer, 2020	Valencia, Spain	RCT	Placebo	29	Probiotic	30	MagNa Pure LC DNA Isolation kit	Universal	Universal	MiSeq	16S V3‐V4	PRJNA629283
Schulz‐Weidner, 2021	Giessen, Germany	Cross‐Sectional	ECC	20	ECC	20	DNeasy PowerSoil Pro Kit protocol	F515 (5’‐GTGCCAGCMGCCGCGG‐3′)	R806 (3′‐TAATCTWTGGGVHCATCAG‐5′)	MiSeq	16S V4	PRJNA731066

*Note*: Key features of each individual study incorporated into meta‐analysis. Including: Geographical location, study design, controls and cases used, DNA extraction methods, 16S primers used, sequencing platform and region of 16S rRNA amplified.

### Assessment of alpha and beta diversity

**FIGURE 2:**
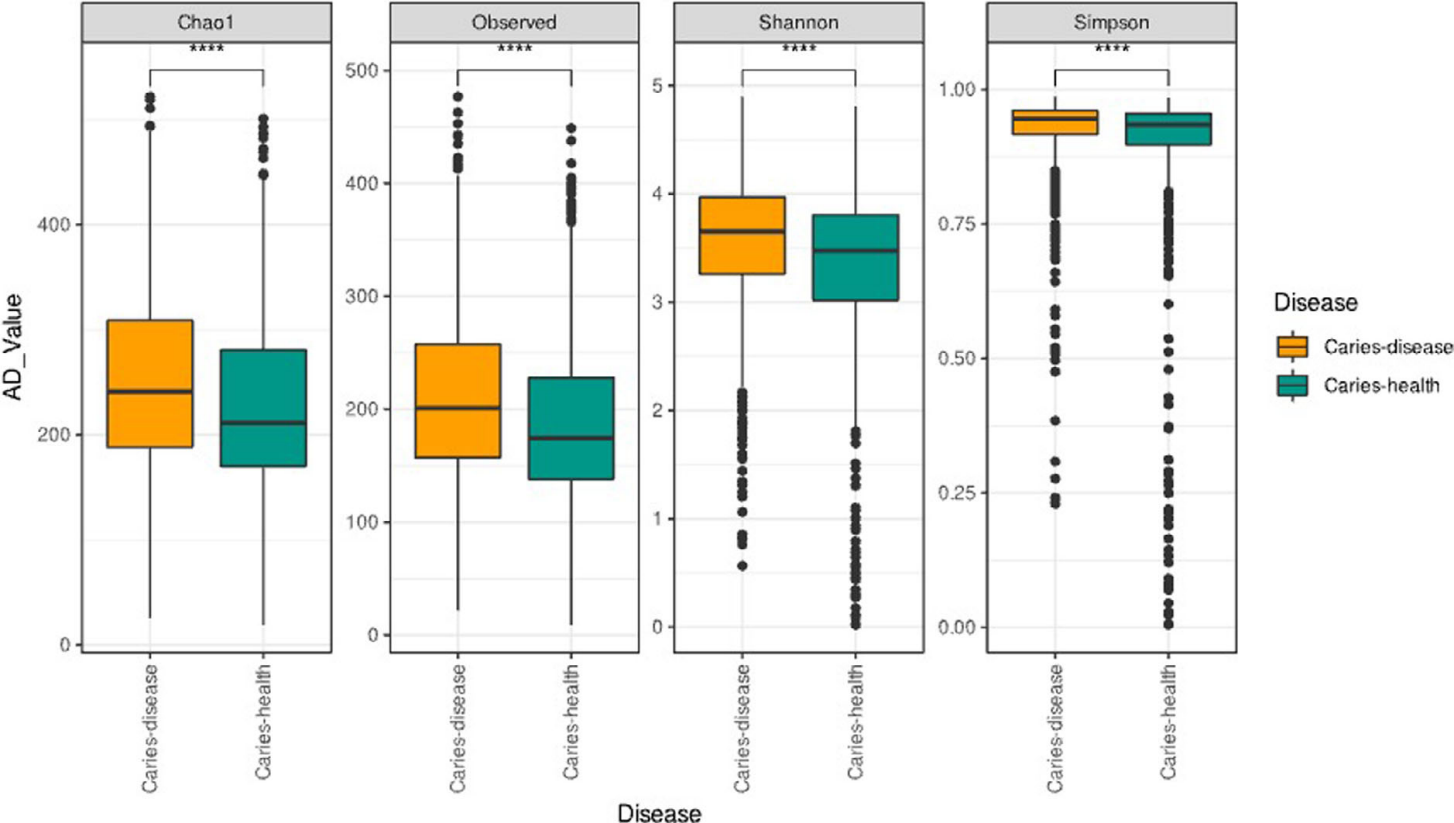
Boxplots of alpha diversity indexes reflect bacterial abundance and evenness. Chao1, observed, Shannon and Simpson diversity indexes are shown for each comparison. Higher values in the chao observed and Shannon indexes indicate a higher diversity in the microbiota. The higher the value of the Simpson index the lower the overall diversity of the microbiota. Boxplots depict the median and upper and lower quartiles of the samples grouped by healthy or caries diseased individuals.

Sample data were processed and removed if less than 1000 total passed reads before plotting to show the average reads per sample per study. Alpha diversity analysis was conducted using the observed, Chao1, Shannon and Simpson diversity indexes on the combined sample set (Figure 2). Data were plotted and analysed according to whether the sample was classified as being derived from a caries or healthy patient sample. Median values across diversity indices were shown to be significantly higher in average alpha diversity across all measures of diversity, abundance and richness for caries when compared to health (*p* < 0.001). Next, we separated the sample data to assess potential differences between by childhood *versus* adult caries. This was motivated by the volume of samples identified as taken from children in the cohort data being approximately one third of the total sample population. The Chao1 and Observed diversity indexes show that there was no significant difference between disease and health in adults. However, Shannon diversity index revealed significant differences (*p* < 0.001) between caries and health in adults and children. While the matrices were successful in determining significance between disease and health, there were less significant differences between adult and childhood organism diversity in the Chao1 (*p* < 0.05) and Shannon (*p* < 0.01). (Figure S1).

**FIGURE 3:**
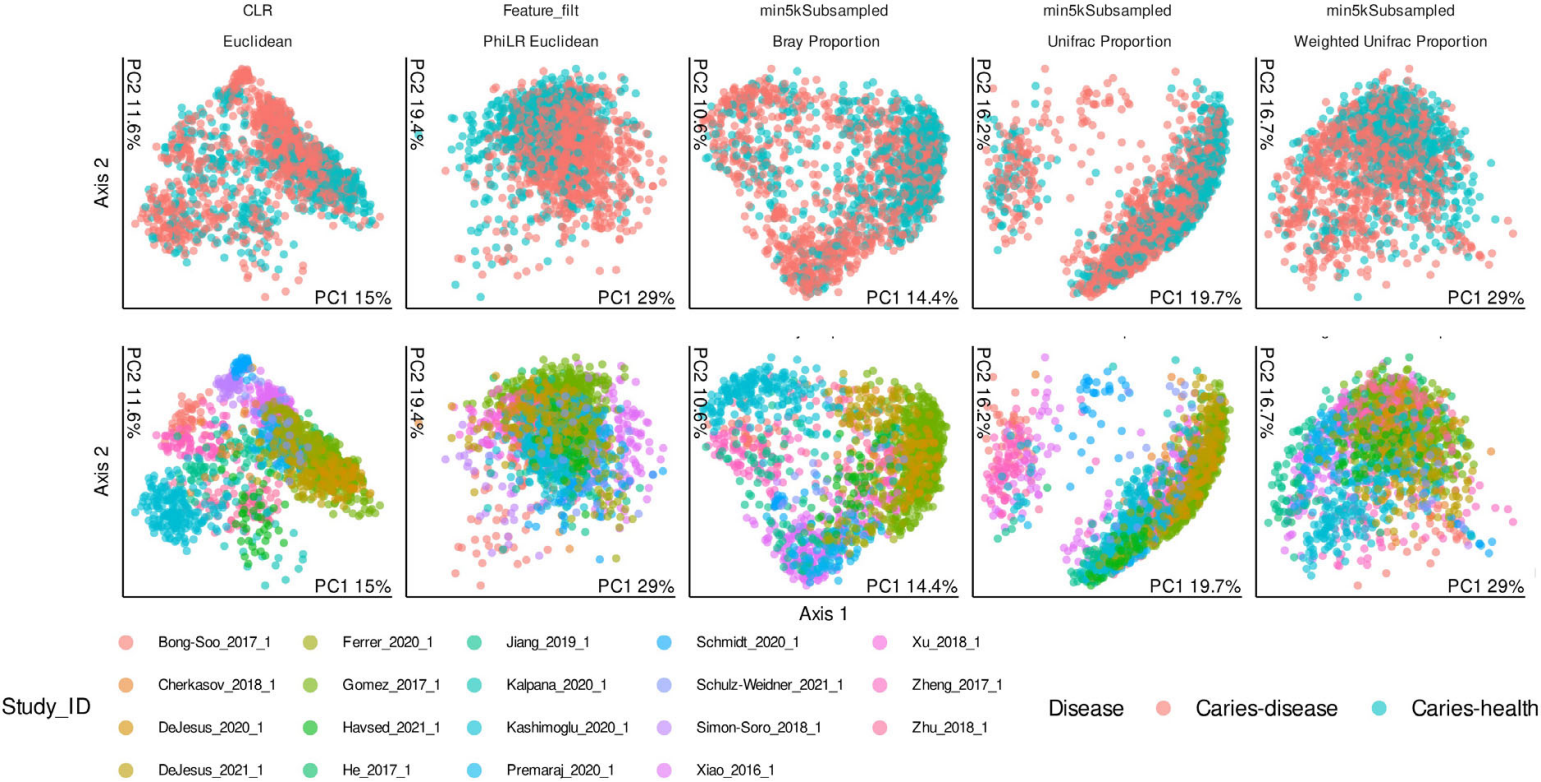
Principal coordinate analysis of caries Oral samples by disease or study identifier. Observed differences in beta‐diversity when comparing sample data from individual microbiome studies (top) or comparing health *vs*. disease (bottom). Sample data taken from individual studies show clear clustering together while distance metrics in health *vs*. disease map an overall poor correlation between identifiable features.

We next utilized visualization of beta diversity to assess the differences in community composition based upon our different parameters through principal coordinates analysis of multiple distance metrics (Figure 3). Data were separated through colour demarcation where colours were assigned to caries (pink) and health samples (blue) in the **top** panel, and individual studies in the **bottom** panel. As noted by Bisanz *et al* (2019), due to matrix sparcity, phylogeny aware matrix was used, and visual clustering was observed (19). Specifically, Unifrac, Bray–Curtis and Euclidean, based on CLR normalized data, diversity matrices observed clear clustering of samples from the same study when analysing study‐to‐study diversity. Similar observations were also noted in Figure S2 when analysing sequencing region chosen, where clearly defined clustering was observed by sequenced region across all diversity metrics. Statistical Significance was observed in the ADONIS statistical metrics across all diversity matrices (*p* < 0.001) as described in Table S1.

### Assessment of diagnostic capability of study metadata

**FIGURE 4:**
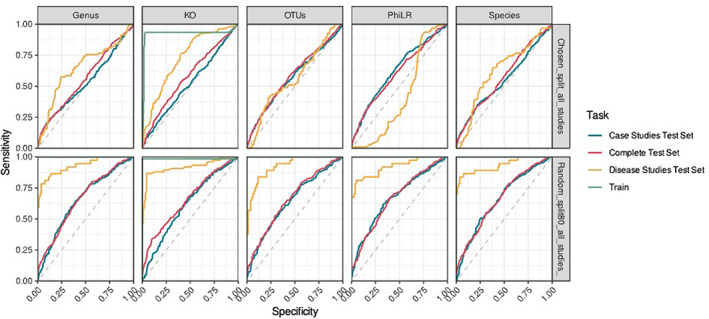
Receiver operator curves from random Forest based upon feature tables summarized to OTU, genus, species, PhiLR and KO (KEGG Orthology). Sample differentiation determined by area over curve compared for each functional group, where a value closer to 1.00 represents a clear ability to predict caries status. Data were trained using 3 case‐controlled studies compared to all remaining studies (top) and an 80/20 split of all available sample data to training dataset (bottom).

Following this, we aimed to evaluate the predictability of caries disease state using receiver operating characteristic curves (ROC) derived from random forest classifiers across Genus, Kegg Orthology (KO) assignment, OTUs, Phylogenetic Isometric Log‐Ratio Transform (PhILR) and Species. We initially adapted a training set from a subset of 4 high‐impact studies out of the 20 case‐controlled sample set (Figure 4). We demonstrated that the ‘disease only’ study test set was the only one found to be predictable in the KO group, with an approximate area under the curve (AUC) of 0.75. We observed marginal improvement of the predictability of health and disease in the complete test data set when applying a 80/20 train‐test split.

### Recurrent metabolic pathways in study metadata

**FIGURE 5:**
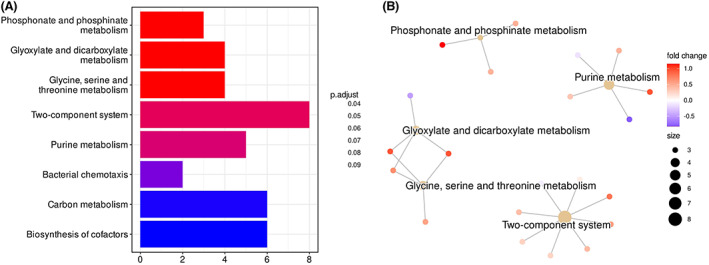
Kegg Orthology mapping of biological pathways. 140 features chosen by cross‐validation of the random forest training set. KEGG ontology over representation analysis was performed within clusterprofiler. The most overenriched pathways are shown. Number of kegg orthology features derived from picrust and the coloured by their adjusted p‐value (A). Pathways are also shown in relation to one another with the feature colourized to indicated whether more represented in disease (red) or more represented in health (blue) (B).

Next, we observed biological pathways associated with cohort microbiome data assessment of KO (Figure 5). In this we found the highest number of distinct pathways that were associated with caries to be phosphonate and phosphinate metabolism and glyoxylate and dicarboxylate metabolism (*p.adj* = 0.04), both key processes in fuelling carbohydrate metabolism (33, 34). Conversely, we found carbon metabolism and cofactor biosynthesis to be less significantly associated with caries across the cohort of studies (*p.adj* = 0.09), both of which are essential metabolic pathways associated with cell synthesis (35). The most abundant feature observed was that of the two‐component regulatory system, which is associated with a response to environmental factors, as well as an upregulation in microbiological virulence factors (36, 37).

### Organisms of interest in caries and health

**FIGURE 6:**
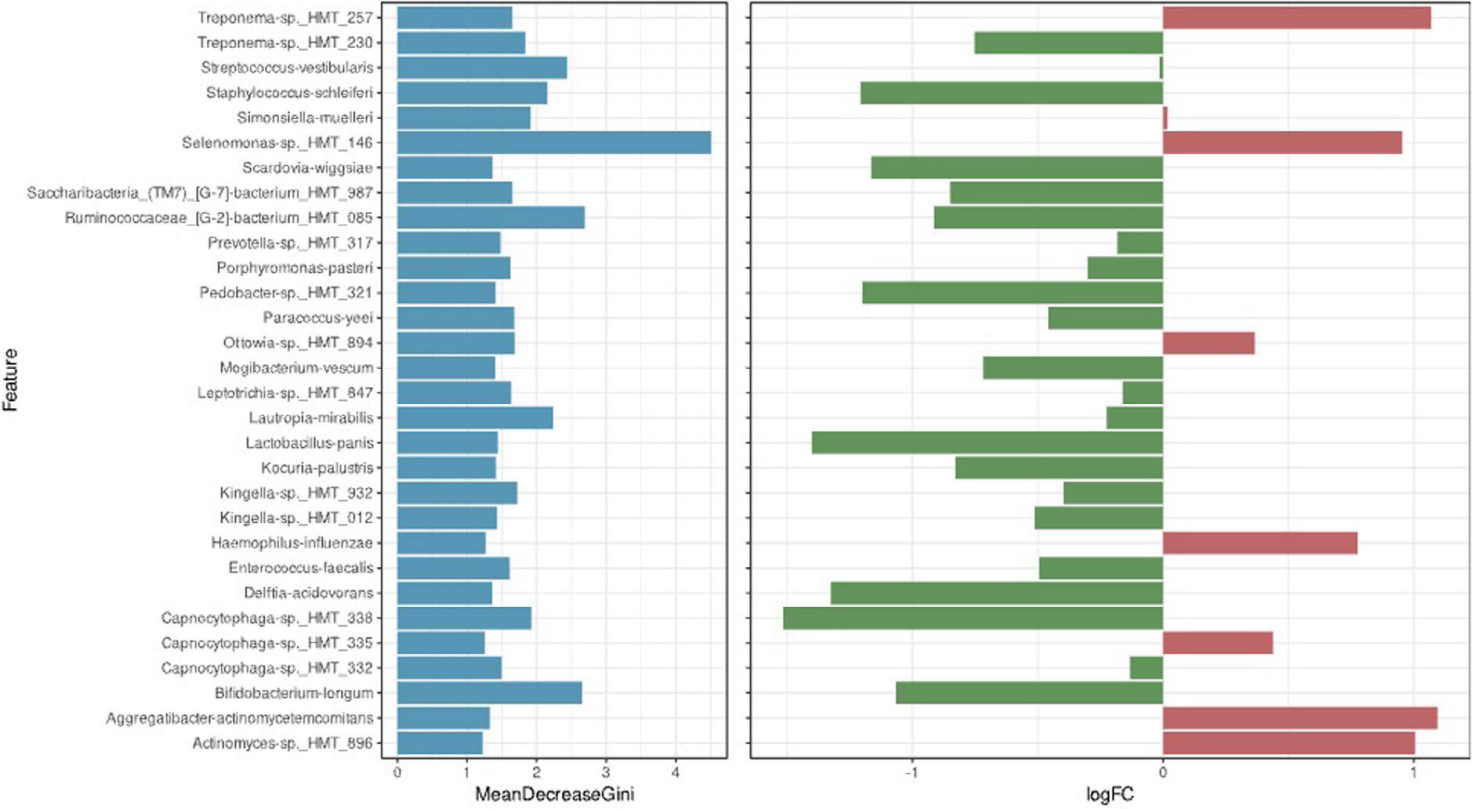
Differential microbial distribution across all studies when comparing caries and healthy microbiomes. Bacterial species selected by mean decrease in accuracy of the Gini coefficient in the random forest classifier for determining differences between the two groups. The corresponding differential abundance of each of the features is represented by its log2 fold change between caries and health. Organisms denoted in green are more important in prediction of carious microbiome while those in red are more predictive of ‘healthy’ microbiome.

When assessing differential microbial distribution across all studies (Figure 6), we noted that a mean decrease in accuracy was most obvious in *Capnocytophagia* spp HMT‐338, *Lactobacillus panis, Treponema* spp. HMT‐230, *Bifidobacterium longum, Delftia acidovorans* and *Staphylococcus schlieferi* inferring that these organisms are important when predicting between ‘health’ and disease groups. Conversely, *Selenomonas* spp HMT‐146., *Agregatibacter actinomycetemcomitans*, *Actinomyces* spp HMT‐896 and *Treponema* spp HMT‐257 observed a higher log_2_ fold change in prediction of the carious microbiome.

### Summarizing the individual predictability of cohort studies

**FIGURE 7:**
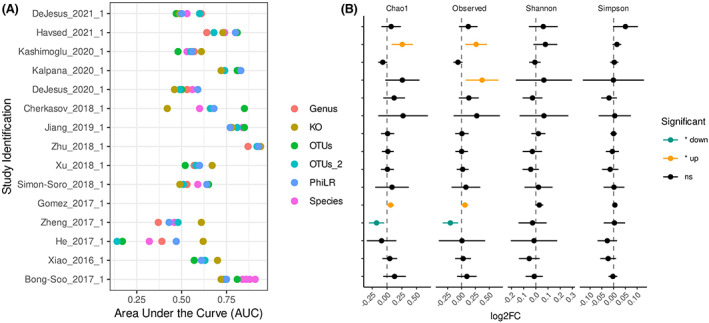
Comparisons of the microbiome in case‐controlled studies. Area under the curve was calculated for the performance of the random forest classifier in each of the case‐controlled studies between the caries and health groups. Random forest classification was performed on feature tables at OTU, genus and species level. Additionally, it was performed on Kegg Orthology derived from picrust and phylogenetic transformed data (PhILR). For each study, the diversity indexes Chao1, Shannon and Simpson were calculated and are represented as a Log2 fold change between disease and health. Welch's t‐test was performed and points are coloured when the significance = *p* < 0.05.

Finally, we calculated area under the curve using random forest classifiers for 15 individual studies to identify their predictive capacity for determining caries microbiome (Figure 7 **)**. 3 studies were removed from the initial cohort as they did not contain comparisons between health and disease, either being health only or disease only. Amongst these classifiers, studies performed by Bong‐Soo *et al* (2017), Zhu *et al* (2018) and Jiang *et al* (2019) (38, 39, 40) were found to perform the best as differentiators between health and disease, with mean AUC scores over 0.75 (Figure 7A). No correlation was observed between cohorts to determine which diversity metric was the most accurate predictor of disease. Additionally, we calculated alpha diversity indices denoted by Log_2_ fold change between disease and health across these studies (Figure 7B). A significant increase in Chao1 richness was observed in 3 of the 15 studies, while observed abundance was also increased significantly (*p* < 0.05) in the same 3 studies, as well as pooled studies. Overall, there was little significance to be observed in alpha diversity metrics across the sample study groups.

## DISCUSSION

The microbiome has been closely linked to human health and disease in many contexts and continues to gain ground in terms of accessibility through technological breakthroughs, economic viability and higher throughput sample processing (41, 42, 43). In the oral cavity, the concept of ecological changes to the environment has been examined in detail as a fundamental starting point for hypotheses surrounding disease development (44, 45, 46). Indeed, as the microbiome has been explored in the context of oral healthcare, many positive implications have been found to support defined taxonomic differences in health and disease in periodontitis (47, 48). However, it is largely understood that development of the disease microbiome in periodontitis remains ecologically distinct from caries due to key differences in subgingival sample sites and mounting immunological pressure from the host response (49, 50).

In this study, we sought to take a broader contextual view of caries and examine multiple studies related to caries microbiome research to determine what, if any, key microbiological markers could be used to discern whether there was adistinct cariogenic microbiome profile. We hypothesized that individual studies lack power to accurately describe whether a specific microbiome profile is associated with caries, and we can answer this through amalgamation of publicly available study data. To begin with, we examined the literature on a wider scale, using study selection and data extraction techniques derived from systematic reviews and meta‐analytic microbiome research, comparable to those studies carried out by Guerra *et al* (2018) and Romano *et al* (2021) (48, 51). This approach ensured a stringent and non‐biased collection of study data that could be used to inform our analytics of the landscape of caries sequencing research. This initial examination of the literature, as summarized in Figure 1B, outlined a lack of uniformity in study methods and drew our attention to the wide differentiation between many different, and potentially severely impactful, metrics across the available datasets. Indeed, work carried out by Scherz *et al* (2021) has highlighted the need for quality control in microbiome‐driven research, and a call for uniformity across methodologies to ensure the collectively available pool of knowledge surrounding microbiome research, not only for oral health, is as accurate, reproducible and representative as possible (52). In addition to this, of the initial 73 studies we determined to meet the minimum criteria for data collection, worryingly only 30% of these were found to have sufficient access to data that would permit meta‐analysis. While there is a trend towards better inclusion rates for sample data, as peer‐review requirements for data upload have improved over the last decade, there is still an obvious gap in the standard of uploaded data in terms of its completion and consistency, as highlighted by Langille *et al* (2018) (17). Indeed, the implementation of protocols such as ‘Strengthening The Organization and Reporting of Microbiome Studies’ (STORMS) further emphasizes our call for consistent and standardized reporting of microbiome data in oral research (16). Indeed, little is done to address the potentially multifactorial impact of circumstances surrounding environment and healthcare as would be covered by Social Determinants of Health (SDH) (53).

Like landmark meta‐analysis of microbiome data that have been performed on the gut microbiome, the data utilized were accessed through the PubMed® database (18).

It should be stated, however, that as we have limited published studies to those only obtainable *via* PubMed®, this leaves a wide array of published material available *via* other archives to be interrogated. We also acknowledge that studies excluded from pre‐2012 may have provided more data but we do not believe would have inferred any different outcome in terms of standardized methodologies.

Despite these barriers, we were, however, able to access and process data from 19 studies. In alpha diversity metrics across these combined studies, we observed significant differences in diversity, richness and abundance across health and caries. This is a significant and novel finding because at an individual study level previous data sets from within some of the cohorts were unable to report any significant differences in their populations (40, 54, 55, 56, 57). This would immediately imply the value in collaborative combination of study cohorts in predicting disease outcomes, while also indicating the benefit of larger cohort sizes within individual studies as a potential compensatory mechanism. Moreover, while some studies have indicated that age can be an important factor in caries status (58, 59), and while we found some significant differentiation in alpha diversity between childhood and adult caries and health in the cohort metadata, the comparisons of interest between health and disease in each sub‐group also showed significant differences. We also aimed to examine other confounding factors that could explain some negative findings to individual studies. We therefore assessed the impact that the sequencing region played amongst the combined study cohort. As stated previously, it has been well defined in the literature that the selected 16S rRNA region amplicon can have a profound impact on the amplification of select taxa within the sample (60, 61). Indeed, our own findings indicated clear clustering amongst amplicon region, while showing minimal definable clustering between health and disease across beta diversity matrices.

We next applied area under the receiver operator curve (ROC) analysis based on random forest feature tables to investigate the ability to predict caries. This was accurate across each feature parameters when disease‐specific studies were separated but was unable to reliably predict caries status on the full combined cohort. This is reflective of results found by Zhang *et al* (2020), where prediction of caries disease was not achievable from tested genera (62). Notably, this conflicts with studies conducted by Teng *et al* (2015), Zhu *et al* (2018) and Wu *et al* (2021) (39, 63, 64), where they have reported reliable prediction of caries onset through microbiome analysis. However, it should be stated that multifactorial differences are visible in the ability to readily predict disease outcome. For instance, It has been reported that onset of caries in a longitudinal setting is independent of genetic factors, while being impacted by acquired behaviours, and that predictability of disease is compounded by these factors as well as other microbial elements, such as the prevalence of *Candida albicans* (64, 65, 66). This is a glaring omission in all of these studies, and the inclusion of mycobiome analysis may be a helpful tool to support our understanding of caries‐related plaque. Indeed, while our initial analytic intent was to encompass the mycobiome and fungal‐related research, we found only 1 fungal mycobiome study that we were able to shortlist from available publications to carry forward for robust analysis. Interestingly, our analysis of KO elements provides valuable insight into the potential mapping of metabolic profile to disease prediction given the observed trend from common baseline metabolic pathways to those more abundant in virulence and sugar metabolism which are key factors in degradation of caries outlook. Taken together, the use of sophisticated bioinformatic tools from profiled microbiomes and mycobiomes will enhance our ability to predict and manage oral disease with a greater level of confidence. Indeed, we identified *Selenomonas* spp HMT‐146., *Aggregatibacter actinomycetemcomitans*, *Actinomyces* spp HMT‐896 and *Treponema* spp HMT‐257 as prevalent within the cohort data as markers of caries. These organisms have been routinely isolated in previous studies investigating caries microbiome, while also being linked to cariogenesis and abundance in high sucrose environments (67, 68, 69). This, again, emphasizes the essential need to assess environmental factors in determination of caries outcome alongside microbiome‐driven research to predict a definitive outcome. Additionally, the recurrence of *Treponema* species as potential differentiators for both health and caries continue to drive the emphasis that being able to reliably speciate within microbiome analysis is essential in disease profiling (70). Finally, the lack of representation of *Streptococcus*, particularly *Streptococcus mutans*, as an impactful predictor of caries does not mesh with the conventional understanding of the disease profile and perhaps encourages the need to move away from convention and pre‐disposed bias in the light of new methodologies and data.

Finally, we analysed the study cohort data on an individual level, using AUC scores to determine predictability of caries on a case‐by‐case basis. These individual studies were then investigated for correlating factors in study design which may have provided insight into the ‘best‐practice’ for achieving a microbiota‐specific diagnosis. We were unable, however, to determine any obvious overlapping factors in study design which could be attributed to this but may be resultant from factors that were not readily shared in the study publication. The inconsistencies that are visible from these analyses also have wider‐reaching implications in the reliability of producing pre‐clinical biofilm models that can accurately provide a basis for experimentation on caries *in vitro*, such as those previously published by our own group (71, 72). Indeed, the future direction for the development and testing of new oral hygiene therapeutics may be in the use of undefined biofilm consortia that can be easily analysed using cheaper and more reliable sequencing technologies.

## CONCLUSIONS

As the field of microbiome research continues to expand at a rapid pace, and the volume of microbiome data that is generated increased, then we should be mindful of a potential trend towards continuing to develop non‐standardized methodologies or insufficiently differentiated microbiome analyses which, as previously indicated by Holman *et al* (2015), may provide room for bias (73). This is of relevance when considering the sequencing region selected for analysis. In addition, we re‐iterate the need for larger volumes of samples being sequenced to increase the relevance of individual studies being generated as a reliable metric for microbiome characterization. Indeed, through advancement of available sequencing technologies and techniques which can analyse the entire 16S gene in a cost and time‐effective manner, we believe that a shift towards broad standardization of methods is attainable in the near future (74, 75).

## Supporting information


**Figure S1** **Further breakdown of alpha diversity examining adult Vs. childhood caries in health and disease.** Chao1, Observed, Shannon and Simpson diversity indexes are shown for each comparison. Lower median values for childhood samples in Chao1, Observed and Shannon indexes reveal lower microbial diversity in healthy childhood samples when compared with all other samples.

**Figure S2** **Principal Coordinate Analysis of Caries Oral Samples depicting beta diversity of microbial population.** (**A)** Amplified 16S sequence region and (**B)** Adult and childhood health and disease. Overall, clustering of samples based on sequence region is most prominently identifiable when observed using Euclidian, Bray‐Curtis and Unifrac similarity metrics. When comparing health and disease, sample clustering is only marginally distinguishable using Euclidian metrics.

Figure S3

**Table S1** Table displaying results from ADONIS testing for the entire dataset

Table S2
