# Supplementary figures and images for: Meta‐analysis of caries microbiome studies can improve upon disease prediction outcomes
Source: APMIS. 2022 Sep 20;130(12):763–77. doi: 10.1111/apm.13272 (PMC9825849; doi:10.1111/apm.13272)

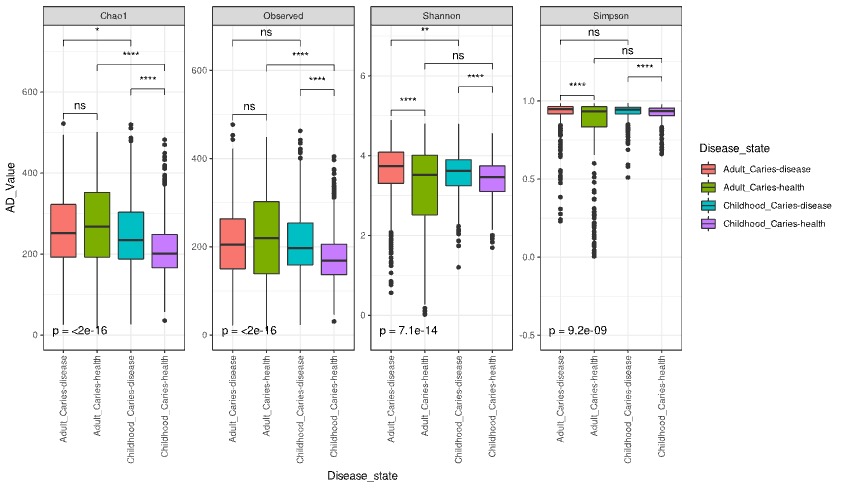

Supplement: Supplementary file 1 — Figure S1 Further breakdown of alpha diversity examining adult Vs. childhood caries in health and disease. Chao1, Observed, Shannon and Simpson diversity indexes are shown for each comparison. Lower median values for childhood samples in Chao1, Observed and Shannon indexes reveal lower microbial diversity in healthy childhood samples when compared with all other samples. [file APM-130-763-s004.tif]

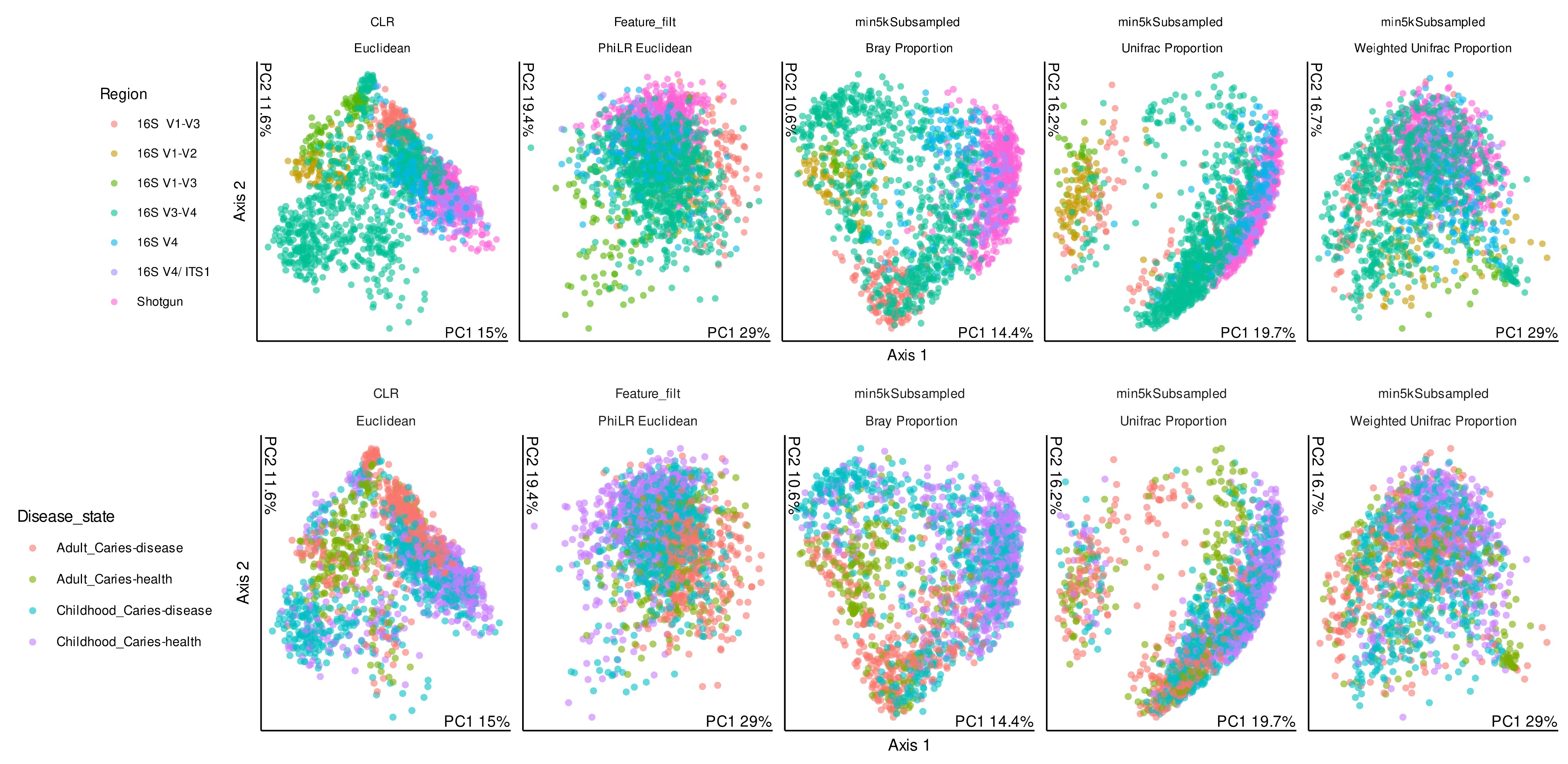

Supplement: Supplementary file 2 — Figure S2 Principal Coordinate Analysis of Caries Oral Samples depicting beta diversity of microbial population. (A) Amplified 16S sequence region and (B) Adult and childhood health and disease. Overall, clustering of samples based on sequence region is most prominently identifiable when observed using Euclidian, Bray‐Curtis and Unifrac similarity metrics. When comparing health and disease, sample clustering is only marginally distinguishable using Euclidian metrics. [file APM-130-763-s001.tif]

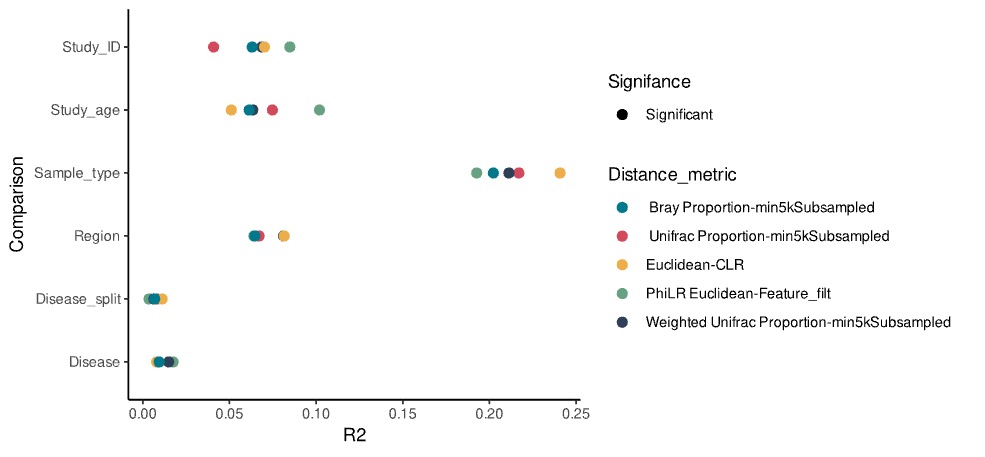

Supplement: Supplementary file 3 — Figure S3 [file APM-130-763-s005.tif]
